# Assessing the Adherence to Safety Protocol Among Personnel Working in the Dental Radiology Department: A Qualitative Cross-Sectional Study

**DOI:** 10.7759/cureus.59502

**Published:** 2024-05-02

**Authors:** Aarati S Panchbhai, Prasanna R Sonar

**Affiliations:** 1 Dentistry, Sharad Pawar Dental College and Hospital, Datta Meghe Institute of Higher Education and Research, Wardha, IND; 2 Oral Medicine and Radiology, Sharad Pawar Dental College and Hospital, Datta Meghe Institute of Higher Education and Research, Wardha, IND

**Keywords:** occupatiopnal health, occupational personnel, safety protocol, radiation, exposure

## Abstract

Background: Implementing recommended radiation safety protocols in the radiology departments is crucial for the safety of the working personnel, patients, and environment. Similarly, adherence and continuous monitoring are essential, and few studies have been conducted in dental radiology settings regarding the occupational safety of employees. Hence, the present study was carried out to assess the knowledge and adherence of personnel to safety protocol in the dental radiology department.

Materials and methods: The questionnaire-based study was conducted in a dental institute involving the relevant staff and students, consisting of 25 participants including radiographers (a technician, faculty, and postgraduates) and auxiliary staff. The 14-element questionnaire with questions on radiation exposure, hazards, safety protocols, protective devices, and infrastructure was given to study participants, and data was obtained.

Results: Overall, the responses to the questionnaire were favorable in terms of awareness or knowledge of participants about radiation protection and protection devices. Notably, 71.4 % (15) of the participants agreed that they consistently wear PPDs, and 86.5% (18) agreed that the radiology operatory is well-equipped concerning radiation safety measures. On the whole, the responses to the questionnaire were encouraging from the auxiliary staff in terms of radiation protection awareness.

Conclusion: The perception of study participants provides valuable insight to enhance the adherence to radiation safety protocol in the institutes. The study demonstrated that most participants revealed adequate knowledge of radiation exposure hazards, the need for personnel protection, and adherence to radiation safety protocol. This study would serve as the pilot project and may provide a platform for further multicenter studies to be carried out.

## Introduction

The knowledge of dental health professionals about radiation safety-related guidelines is crucial. The imaging field has become very expansive with numerous advancements in imaging protocols. Subsequently, awareness and knowledge concerning radiation hazards, protection measures, dosimetry usage, and infrastructure have become a prerequisite, and several challenges with radiation protection and safety culture in radiology departments need to be addressed [1-5]. All personnel in the radiology department need protective equipment and proper monitoring while working, and one of the tools to monitor personnel exposure to radiation would be personal dosimeter badges. Additionally, waste disposal of hazardous material in the radiology department is a separate domain to deal with [1-6]. 

The International Commission on Radiological Protection (ICRP), the National Council on Radiation Protection and Measurements (NCRP), and the Atomic Energy Regulatory Board (AERB) provide guidelines about permissible doses, equipment, and working protocols for occupational and nonoccupational personnel. The rules and regulations about radiation safety norms are to be followed by all the dental operatories in India. According to the As Low As Reasonably Achievable (ALARA) principle, the magnitude of individual doses, the number of people exposed, and the likelihood of gaining exposure should be kept as ALARA. No practice relating to radiation exposure should be approved unless it produces a sufficient benefit to the exposed individual. Achieving optimum quality and personnel safety is the target of every healthcare institute. Radiation can cause damage to human tissue indirectly or directly if the institute or working personnel does not adhere to the guidelines or follow the recommended safety protocols; hence, carefully crafted strategies are essential in a dental hospital to create a positive safety culture [1,5-13]. 

Rationale of the study

Work safety is the basic right of every employee. The knowledge of dentists about radiation protection techniques was very poor, and they should implement proper radiation protection techniques and guidelines [2]. Additionally, there should be concerns about secondary radiation, and means of protection should be in place to prevent X-ray radiation from reaching out of the X-ray room in the department. In a healthcare setting, a lot of attention is paid to patient safety with a minimum focus on employee safety and work culture. The working personnel in the radiology department may get exposed to harmful factors such as radiation exposure, exposure to body fluids, needle pricks, and poor ergonomics so the strategies to prevent and reduce these should be in place in every hospital with surveillance. Not only the patient getting exposed but the occupational and nonoccupational people are getting exposed to radiation. These include faculties, technicians, postgraduates, nurses, attendants, and so on. Precautionary safety measures are needed for all of them. For improvement in any healthcare facility, understanding the perception of the related personnel is critical to provide valuable insight to enhance service outcomes. Very few studies are conducted in dental healthcare settings regarding occupational safety in the radiology department; therefore, the literature is scarce. Given this, the present study was conducted to evaluate awareness about guidelines and safety protocols to be followed by the personnel working in the dental radiology department and its implementation.

Objectives of the study

This study aims to assess the knowledge of the personnel about the need for radiation protection in the dental radiology department using the Likert scale and to assess the adherence of the personnel to the radiation safety protocol in the department using a rating scale.

## Materials and methods

The present ethics committee-approved study (Institutional Ethics Committee, DMIHER(DU)/IEC/2023/1199) was conducted in the Department of Oral Medicine and Radiology in a dental college. The sample was recruited from the radiology department, and the 25 participants included postgraduate students and teaching and nonteaching staff working in the radiology department. The study protocol was explained to the participants, and the consent was obtained. The participant’s knowledge about the need for radiation protection and safety measures and adherence to radiation safety protocol in the dental radiology department was assessed using a questionnaire. The responses were analyzed, and quantitative data was obtained.

The inclusion criteria included the following: All occupational people getting exposed while there is a radiography procedure, all faculties and postgraduates (PGs) working in the dental radiology department, and all the nonteaching staff working in the dental radiology department. The exclusion criteria included all faculty and PGs not working in the radiology department or not contributing to the radiographic examinations.

The questionnaires were given to the participants to obtain data about their knowledge about radiation protection and its implementation. The information about demographic data, such as age and gender was collected, followed by details about the study protocol. The close-ended questionnaire consists of 14 elements with various questions about radiation protocol, radiation hazards, protection measures, and safety-related knowledge of technicians, faculty, and postgraduates. The customized questionnaire was prepared and prevalidated by the education unit experts and pretested by piloting before use. The questionnaire was divided into two parts: The first part was about the knowledge/awareness of participants about radiation protection, and the responses were based on options such as "Yes, Neutral, or No." The second part was about the implementation and adherence to safety measures in the radiology department, and the responses were based on a five-point Likert scale ranging from "strongly agree to strongly disagree." Additionally, participants’ rating of radiation protection practices in the dental radiology department was obtained on a 1-5 rating scale. As the nonteaching or auxiliary staff were not contributing to the radiographic examination procedures, a separate questionnaire was given to obtain their perception of radiation protection practices.

**Figure 1:**
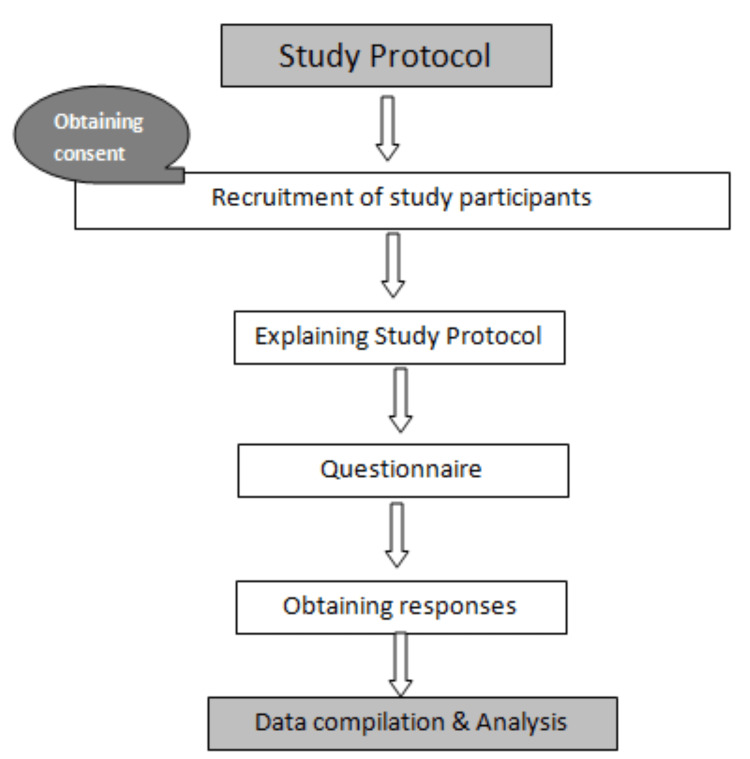
Flowchart of the study protocol

For data analysis, descriptive statistics was done, and the frequency of responses to each item and their percentages were obtained and tabulated. The flowchart of the study protocol is shown in Figure 1.

## Results

The study participants included 21 radiographers, i.e., postgraduates, faculty, and a technician collectively, and four auxiliary staff working in the dental radiology department. The completed questionnaires from 25 patients were collected with a 100% response rate. 

**Figure 2:**
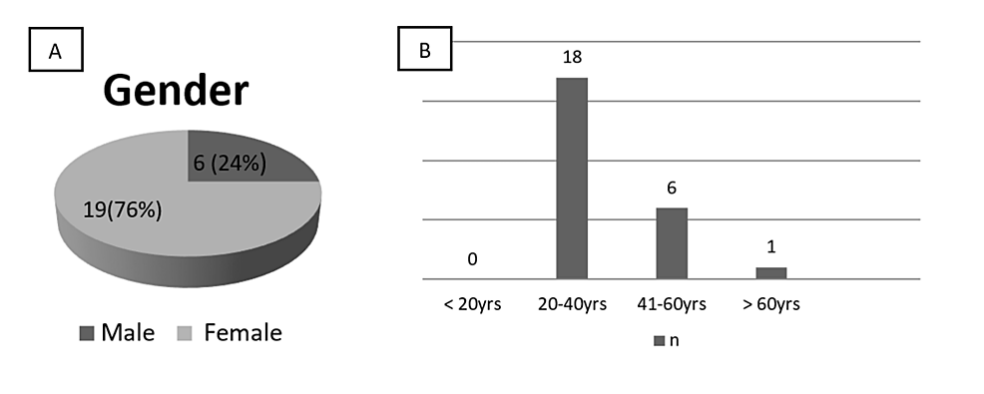
(A) Gender-wise distribution of the participants; (B) age-wise distribution of the participants (yrs, age in years)

Of the total sample of 25, 76% (19) were female and 24% (six) were males. The participants were in the age group of 23 to 63 years, as shown in Figure 2A and Figure 2B. The questionnaire given to the radiographers consisted of 14 elements in two parts. The first part dealt with the knowledge/awareness of participants about radiation hazards and protection, and the second part was about the implementation and adherence to the safety protocol in the radiology department. The questionnaire given to the auxiliary staff had six items for obtaining their perception.

Assessment of the knowledge of personnel about the need for radiation protection

Assessing the knowledge of personnel about the need for radiation protection in the dental radiology department is shown in Table 1. The first part which concerned the knowledge/awareness of participants about the need for radiation protection consisted of seven elements based on "Yes/Neutral/No" options. For the items, "Are you aware of the need for radiation safety measures," "Have you attended any training on radiation protection measures," and "Are you aware of radiation dosimeter and its use," the response of 100% of the participants was "Yes." For awareness of the ALARA principle, 19 of 21 (90.1%) participants replied yes, one was neutral, and one participant replied "No." For the question "Do you know appropriate radiation exposure parameters," 20 participants (95.5%) replied "Yes," while one response was neutral. All the participants knew about personal protective devices (PPDs), while the response was suboptimal toward awareness of the ICRP, NCRP, and AERB recommendations. Overall, the responses to the questionnaire were favorable in terms of the awareness or knowledge of the participants about the need for radiation protection and protection devices.

Assessment of the adherence of personnel to radiation safety protocol

**Table 1:** Knowledge of the participants about the need for radiation protection in the dental radiology department (N = 21)

Sn	Items	Yes		Neutral		No	
	QUESTIONNAIRE	n	%	n	%	n	%
1.	Are you aware of the need for radiation safety measures	21	100				
2.	Have you attended any orientation/training on radiation protection measures	21	100				
3.	Are you aware of the radiation dosimeter and its purpose	21	100				
4.	Are you aware of as low as reasonably achievable (ALARA) principle	19	90.4	1	4.76	1	4.76
5.	Do you have knowledge about appropriate radiation exposure parameters	20	95.2	1	4.76		
6	Do you have knowledge about personal protective device (PPD)	21	100				
7	Are you aware of the radiation council's recommendations	16	85.7	3	14.2	2	9.52

Assessing adherence of personnel to radiation safety protocol in the dental radiology department is shown in Table 2. The second part about the implementation and adherence of the safety measures in the radiology department has seven elements based on a five-point Likert scale ranging from "strongly agree to strongly disagree." For the item "You use PPDs/radiation safety measures, i.e., wearing of lead aprons, thyroid shields, and lead goggles consistently," the responses of 19 out of 21, i.e., 86.5% of the participants, were in agreement and 9.52% of participants were neutral. On asking, "whether the radiology operatory is built as per established guidelines of infrastructure," 71.3% of the participants agreed to this, and six, i.e., 28.4% responses, were neutral. For the item "Radiology operatory is well-equipped in regards to radiation safety" measures that 100% of the responses were in agreement. For the item "Whether proper measures are in place for waste disposal in the radiology department," 19 out of 21, i.e., 92.5% of the participants, agreed to this, while 9.52% of participants were not aware of this. Most of the participants (90.2%) wore radiation dosimeters consistently when in the radiology department, while five participants were neutral to following the position-distance rule while taking intraoral radiographs. Most of the participants (90.2%) agreed that maintenance and quality assurance checks are done periodically in the department. On the whole, the responses to the questionnaire were encouraging in terms of implementation and adherence to radiation safety protocols in the dental radiology department.

**Table 2:** Adherence of the participants to radiation safety protocol in the dental radiology department (N = 21) PPDs: Personal protective devices; SD: standard deviation

Sn	Items	SA		S		N		SD		D	
	QUESTIONNAIRE	n	%	n	%	n	%	n	%	n	%
1	You wear a radiation dosimeter consistently when in the radiology department	10	47.6	9	38.09	2	9.52				
2	You use PPDs, i.e., lead aprons, thyroid shields, lead goggles, etc., while taking a radiograph	15	71.4	6	28.5						
3	You follow position-distance rule while taking an intraoral radiograph	2	9.52	10	47.6	5	23.8				
4	The radiology operatory is built as per established guidelines for infrastructure	5	23.8	10	47.6	6	28.5				
5	Radiology operatory is well-equipped in regards to radiation safety measures	18	85.7	3	14.2						
6	Periodic maintenance and quality assurance checks are done in the department	2	9.52	17	80.9	2	9.52				
7	Are the proper waste disposal measures in place in the radiology department (processing solution, lead foil, and film packet wrapper disposal)	5	23.8	14	66.6	2	9.52				

Rating scale about radiation protection practices

Additionally, 21 participants’ rating about radiation protection practices in the dental radiology department was obtained on a scale of 1-5 (1, poor; 2, average; 3, fair; 4, satisfactory; 5, good). The participants were asked to rate on a scale of 1-5 to obtain their perception. The response rate was 100%, and the responses were in the range of 3-5 on the rating scale.

Awareness of auxiliary staff about radiation protection practices

A separate questionnaire was given to four nonteaching or auxiliary staff working in the radiology department to obtain their perception of radiation protection practices. The questionnaire had six elements based on "Yes/Neutral/No" options. For the first item "Are you aware of the need for radiation safety measures," the response of 100% of the participants was "yes." For the item "Have you attended any orientation or training on radiation protection measures," all four (100%) participants replied "Yes" though the degree of assimilation may differ in them on asking, "whether the radiology operatory is built as per established guidelines of infrastructure," two responses were neutral to this. For the item "Radiology operatory is well-equipped in regards to radiation safety measures," 100% of responses were "Yes." The two participants agreed that maintenance and quality assurance checks are done periodically in the department, while two were neutral to this. For the item, "Whether proper measures are in place for waste disposal in the radiology department," 75% of the participants replied "Yes," and one was neutral to this as shown in Table 3.

**Table 3:** Knowledge of auxiliary staff about radiation exposure in the dental radiology department (N = 4)

Sn	Items	Yes		Neutral		No	
	QUESTIONNAIRE	n	%	n	%	n	%
1	Are you aware of the need for radiation safety measures	4	100				
2	Have you attended any orientation/training on radiation protection measures	4	100				
3	The radiology operatory is built as per established guidelines for infrastructure	2	50	2	50		
4	Radiology operatory is well-equipped in regards to radiation safety measure	4	100				
5	Periodic maintenance and quality assurance checks are done in the department	2	50	2	50		
6	Are the proper waste disposal measures in place in the radiology department	3	75	1	25		

## Discussion

The study was conducted to evaluate awareness and adherence to safety protocol among personnel working in the dental radiology department. The total sample of 25 included 19 females and six males; thus, the sample showed female predilection. The participants were in the age group of 23 to 63 years, the maximum being in the age range of 20-40 years as shown in Figure 2.

The response rate to the questionnaire was 100%. The employees and PGs in the radiology department were found to be well aware of radiation safety measures with 100% responses as "Yes." It was a rewarding factor that 100% of personnel in radiology have attended training on radiation protection measures. As per mandates, all teaching and nonteaching employees should be trained optimistically to handle various hospital issues including radiation operatory as per recommended protocol [1-7].

Overall, the responses to the questionnaire were favorable in terms of awareness/knowledge of participants about radiation hazards, safety protocol, and protection devices. The findings corresponded to a study by Aravind et al. [13], Arnout et al. [14], and Basha et al. [15] and were contradictory to other studies [2,4,5,16-24]. Aravind et al. found the level of knowledge acceptable in their study participants [13]. Booshehri investigated radiation protection awareness and knowledge in dentists to reduce radiation exposure [2]. There was no significant difference between knowledge scores among study participants when compared according to gender, age group, or job experiences. The knowledge of dentists about radiation protection techniques was very poor, and they should implement proper radiation protection techniques and guidelines [2].

Arnout et al. and Basha et al. conducted a study on undergraduates or budding dentists to explore their knowledge of radiation hazards and protection which was found to be in the range of low to moderate [14,15]. According to Abuzaid et al., newly graduated and young radiographers exhibited fewer adherences to radiation protection practices as revealed in the study [6]. The emphasis on continuing dental education programs may augment the knowledge of dental students and dentists [14,15]. In our study, the PG students were included as participants to assess their knowledge and performance seeing that they are very closely associated with working in the radiology department, and their knowledge and subject updates would be essential for effective radiology practice.

The second part of the study was about the implementation and adherence to the safety protocol in the radiology department which was based on a five-point Likert scale ranging from "strongly agree to strongly disagree." On the whole, the responses to the questionnaire were encouraging in terms of adherence to radiation safety protocols in the dental radiology department. This corresponds to a study by Arnout et al. where the implementation was found to be in the level moderate to high [14]. While the implementation of standards for quality care and radiation protection was suboptimal in the study by Jacobs et al. [18], Aravind et al. found that 80.3% of the practitioners had a separate section for radiographic examination in their clinics. Although the level of awareness of general practitioners regarding radiation hazards and safety was found to be acceptable, the implementation of their knowledge concerning patient and personnel safety was inadequate [13].

In general, the implementation of radiation safety protocol encompasses techniques, exposure parameters, equipment, film, processing, safety measures, and quality assurance programs in the radiology department. Inadequate literature is available on the studies conducted in dental institutes [2,14,15,21]. Rather, most of the previous studies were conducted on dental practitioners [4,5,13,17-20,22-25]. Altogether, the studies suggested the need for improvement in adherence to radiation safety protocols in either institutes or dental clinics. To summarize the common observations of the previous studies, the E & F speed films, paralleling technique, and rectangular collimations were frequently used in clinics. Contrarily, few studies revealed the frequent use of the short cone technique and circular collimation [4,22,25]. Yurt et al. observed that 62% of dentists enquired about the pregnancy of female patients before imaging [4].

For film processing, both automatic and manual processing were used, although the manual method was of choice [21,25,26]. The use of photostimulable storage phosphor plates and digital radiography systems was occasional in dental colleges which was mainly for research purposes [21,25]. Lead partitions, thyroid shields, and lead aprons were commonly used protective measures. Few dentists used the position and distance rule correctly for their protection [17,23,27]. Overall, the majority of dentists needed orientation on proper methods, materials, and equipment to minimize the exposure of their patients to unnecessary radiation in dental radiography.

Shahab et al. mentioned that proper waste disposal of the used processing solutions and the lead foils was done by only 1% and 3% of dentists, respectively [17], while Eskandarlou et al. observed the lack of regular quality control and quality assurance programs [21]. The studies emphasized continual teaching and modifications to the curriculum, highlighting radiation safety and practice to help enhance the knowledge and competence of students or newly graduated dentists. Moreover, routine continuing dental education seminars/programs may help enhance the knowledge of dentists for a better approach toward radiation safety measures [5,3-15,25,28].

The physical shielding, duration of radiation exposure, and distance from the radiation source are principal factors in radiation protection which will help in reducing personnel exposure. Using various means in the radiology department would help in adhering these key principles. Preplanning the desired images, increasing the distance between the object and X-ray beam, and using personal protective equipment will greatly be of help in minimizing the radiation exposure.

Diagnostic radiology is considered as the third eye, and its use is escalated in both the medical and dental fields. Thus, the radiology department is one of the significant working departments in hospitals with related concerns. One of the concerns for the personnel working in the radiology department is radiation exposure and related complications. Additionally, attention should be paid to factors such as poor ergonomics and the hazardous waste produced in the radiology department on a day-to-day basis. Although it is difficult to quantify the risk of cancer or genetic mutation from diagnostic exposure in patients and working personnel, it has not been possible to prove the absence of such effects [16]. The low radiation doses may add to the probability of cancer occurrence due to changes in cell DNA. According to Abuzaid et al., radiographers play a major role in conducting radiological examinations and are major determinants in radiation exposure; thus, their practice should always be optimized according to the ALARA principle [6]. It needs to work on AERB and NCRP guidelines that provide rules and regulations for working with radiation safety protocols and limiting radiation dose [1-8,12,14,29-33].

The safety measures in the field of healthcare are mainly patient-focused; most of the previous studies have been done for the evaluation of patient safety. A few number of studies have been done on employee safety in hospitals. This study was mainly focused on employees and PGs working in the dental radiology department for the evaluation of pertinent radiation safety conditions. The study has the limitation that the in-depth perception of participants about each item in the questionnaire could not be assessed. Additionally, the study is limited to one dental institute only. These limitations would need to be covered in future extended study. Nonetheless, strategies to prevent and reduce such detrimental factors can be ensured by conducting regular audits or inspections by radiation safety officers and continuous monitoring by the radiologist in charge. Particularly, considerations are required for enhancing the radiographers’ knowledge of the principles of proper radiation protection practices. In this regard, there is definite scope for sensitization sessions or introductory seminars on radiation safety for occupational personnel. 

## Conclusions

The implementation of recommended radiation safety protocols and practices in the radiology departments is vital for the safety of the working personnel, patients, and environment. By following the radiation safety guidelines, dental professionals can protect themselves and others from the hazards of radiation. Institutes can effectively improve radiation protection through compliance with the established standards of practice using proper materials and equipment. A few number of studies have been done on employee safety in hospitals. The present study revealed the importance of awareness and adherence to radiation safety measures among occupational personnel and the need for more studies to be conducted in this domain. A few number of studies have been done on employee safety in hospitals. A coordinated approach is desired to ensure the implementation of radiation protection measures in radiology departments. This study demonstrated that most of the participants revealed a reasonable knowledge of the need for personnel protection, safety measures, and adherence to radiation safety protocol. This study would serve as the pilot project and may provide a platform for further multicenter studies to be carried out. The periodic training and regular monitoring of occupationally exposed health workers as well as PGs should be mandatory to ensure appropriate compliance with radiation safety regulations.
